# Biochemical Characterization of a Carboxylesterase from the Archaeon *Pyrobaculum* sp. 1860 and a Rational Explanation of Its Substrate Specificity and Thermostability

**DOI:** 10.3390/ijms150916885

**Published:** 2014-09-23

**Authors:** Hua Shao, Li Xu, Yunjun Yan

**Affiliations:** Key Laboratory of Molecular Biophysics of the Ministry of Education, College of Life Science and Technology, Huazhong University of Science and Technology, Wuhan 430074, China; E-Mails: shaohua2011@163.com (H.S.); xuli@hust.edu.cn (L.X.)

**Keywords:** carboxylesterase, thermostability, structure modeling, docking, molecular dynamics

## Abstract

In this work, genome mining was used to identify esterase/lipase genes in the archaeon* Pyrobaculum* sp. 1860. A gene was cloned and functionally expressed in *Escherichia coli* as His-tagged protein. The recombinant enzyme (rP186_1588) was verified by western blotting and peptide mass fingerprinting. Biochemical characterization revealed that rP186_1588 exhibited optimum activity at pH 9.0 and 80 °C towards *p*-nitrophenyl acetate (*K*_m_: 0.35 mM, *k*_cat_: 11.65 s^−1^). Interestingly, the purified rP186_1588 exhibited high thermostability retaining 70% relative activity after incubation at 90 °C for 6 h. Circular dichroism results indicated that rP186_1588 showed slight structure alteration from 60 to 90 °C. Structural modeling showed P186_1588 possessed a typical α/β hydrolase’s fold with the catalytic triad consisting of Ser_97_, Asp_147_ and His_172_, and was further confirmed by site-directed mutagenesis. Comparative molecular simulations at different temperatures (300, 353, 373 and 473 K) revealed that its thermostability was associated with its conformational rigidity. The binding free energy analysis by MM-PBSA method revealed that the van der Waals interaction played a major role in *p*-NP ester binding for P186_1588. Our data provide insights into the molecular structures of this archaeal esterase, and may help to its further protein engineering for industrial applications.

## 1. Introduction

Lipolytic enzymes including carboxylesterases and lipases are members of the α/β hydrolase superfamily [1]. Carboxylesterases (EC 3.1.1.1) catalyze the hydrolysis of water-soluble esters with relatively short fatty acid chains (<10 carbon atoms), while lipases (EC 3.1.1.3) are more active towards water-insoluble esters with long chain fatty acids (≥10 carbon atoms) [2]. Besides the difference in their substrate profiles, lipases also differ from esterases in term of the interfacial activation phenomenon [3]. During the past decades, a large number of lipases/esterases from microbes have been reported. Several bacterial and fungal lipases/esterases have become commercial biocatalysts applied in diverse industries such as food, detergent, flavors, fine chemical, cosmetic, biodiesel and pharmaceuticals [4]. However, lipases/esterases usually fail in the reactions performed under harsh conditions, such as high temperature, pH, salinity, and in the presence of organic solvents [5]. Accordingly, considerable efforts have been devoted to screening lipases/esterases with specific properties from cultivated or uncultivated microbes, and even modifying their structures to improve their catalytic properties via protein engineering [2].

Hyperthermophilic archaea are microorganisms that grow optimally at temperatures between 80 and 110 °C [6]. They are regarded as a valuable resource for thermostable enzymes [7]. Thermal stable enzymes are important for industrial uses since the elevated temperature would help to increase conversion rate and substrate solubility, and to reduce the viscosity of the reaction medium [8,9]. Additionally, thermostable enzymes from archaea are good models for elucidating the mechanism of protein thermostability, which will offer valuable information for redesigning proteins with high thermostability [8,10,11]. Until now, several thermophilic enzymes including amylases [12], xylanases [13], chitinases [14], proteases [15], and DNA polymerases [16] have been characterized from archaea, and some of them have widely used in biotechnological applications. However, few lipases/esterases have been characterized from archaea, which are mainly from the genus *Sulfolobus* [17],* Pyrococcus* [18],* Archaeoglobus* [19],* and Pyrobaculum* [11].

In general, thermal stability of a thermophilic enzyme is attributed to many factors including disulfide bridge, hydrophobic interaction, aromatic interaction, salt bridge, and helix dipole stabilization,* etc.* [6,20]. For different enzymes, the contribution may result from different factors. To date, molecular dynamic (MD) simulation is an effective way to evaluate the factors that govern the thermostability of enzymes [21]. This method can provide great details regarding the motion of individual atoms as a function of time in realistic environments. Comparing the dynamic behaviors of a protein at different temperatures will demonstrate the factors that affect protein thermal tolerance [21]. This approach has been employed to study the thermal stable mechanism of esterase [21], lipase [22], phytase [10], and xylanase [23].

As the number of genomes for archaea is growing, it becomes easy to discover useful archaeal enzymes [7]. *Pyrobaculum* sp. strain 1860 is an anaerobic hyperthermophilic archaeon which was isolated from Lake Fumarolic (84 °C, pH 6.8) in Russia [24]. Besides this strain, genomes of other five members in the genus *Pyrobaculum* have been reported [24]. However, only the carboxylesterase PestE from * Pyrobaculum calidifontis* has been characterized, which displayed optimum temperature at 90 °C and maintained well after 2 h incubation at 100 °C [11]. Therefore, in this study, we used genome mining to identify genes encoding putative esterases/lipases in *Pyrobaculum* sp. 1860. One gene (Uniprot: G7VG08) was cloned and successfully over-expressed in *Escherichia coli* as His-tagged fusion protein. The recombinant protein was then characterized for its catalytic properties including substrate profiles, stability and kinetic behavior. Homology modeling was performed to build the 3D model of this enzyme, and its thermostability was further analyzed by molecular dynamic simulation. Then, the combined docking and MM-PBSA method were applied to characterize its substrate specificity.

## 2. Results and Discussion

### 2.1. Sequence Alignment and Structure Modeling

**Figure 1:**
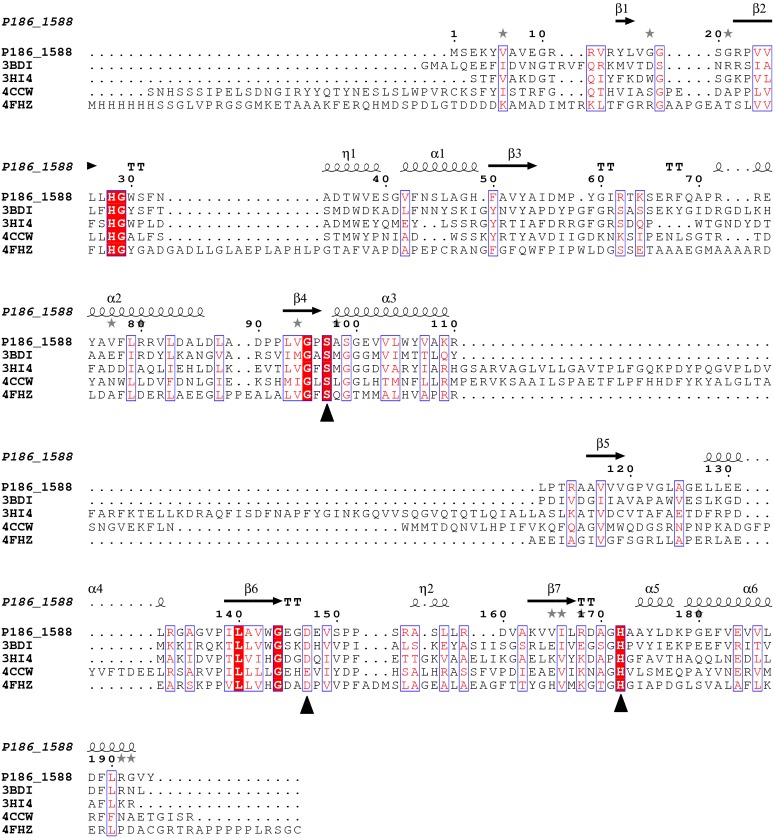
Multiple sequence alignment between P186_1588 and other closely related esterases: 3BDI, an uncharacterized carboxylesterase from *Thermoplasma acidophilum*; 3HI4, a carboxylesterase from *Pseudomonas fluorenscens*; 4CCW, a carboxylesterase from *Bacillus subtilis*; 4FHZ: a carboxylesterase from *Rhodobacter sphaeroides*. The catalytic triad of P186_1588 (Ser_97_, Asp_147_, and His_172_) is marked with filled triangles (■). The alpha helix, beta sheet, random coil and beta turn are identical to α, β, η and T, respectively.

*Pyrobaculum* sp. 1860 is capable of growing in harsh environments (84 °C, pH 6.8), which makes it an attractive source for thermostable enzymes. According to the genome annotation of this strain, only one gene (Uniprot: G7VG08, designated as *P186_1588*) was found to encode a putative carboxylesterase. The gene *P186_1588* consists of 585 bp with GC content of 63.6%, and encodes a protein composed of 194 amino acids with molecular weight and pI calculated to be 21,131 Da and 6.32, respectively. A BLASTP search using the PDB protein database revealed that P186_1588 showed low identity with other carboxylesterases including the uncharacterized carboxylesterase (PDB: 3BDI) from *Thermoplasma acidophilum* (identity: 30%, coverage: 99%); the carboxylesterase (PDB: 3HI4) from *Pseudomonas fluorenscens* DSM 12885 (identity: 27%, coverage: 82%) [25]; the carboxylesterase (PDB: 4CCW) from *Bacillus subtilis* (identity: 29%, coverage: 87%); and the carboxylesterase (PDB: 4FHZ) from *Rhodobacter sphaeroides* (identity: 32%, coverage: 69%) [26], which suggests that P186_1588 might be a novel esterase. Multiple sequence alignment predicted that the catalytic triad of P186_1588 was formed by Ser_97_, Asp_147_ and His_172_ (Figure 1). Generally, the catalytic serine is located in a consensus pentapeptide (G-X-S-X-G). However, Ser_97_ in the predicted catalytic triad situates in a sequence of G-X-S-X-S (Figure 1). Few lipases/esterases have been reported with the serine-containing consensus sequence as G-X-S-X-S [27]. In order to confirm this prediction, Ser_97_, Asp_147_ and His_172_ were mutated into Ala_97_, Asn_147_ and Leu_172_ respectively. The activities of the mutant enzymes were examined with different kinds of *p*-NP esters. None of the mutant proteins showed detectable activity, which confirmed the importance of these residues in the activity of P186_1588.

**Figure 2:**
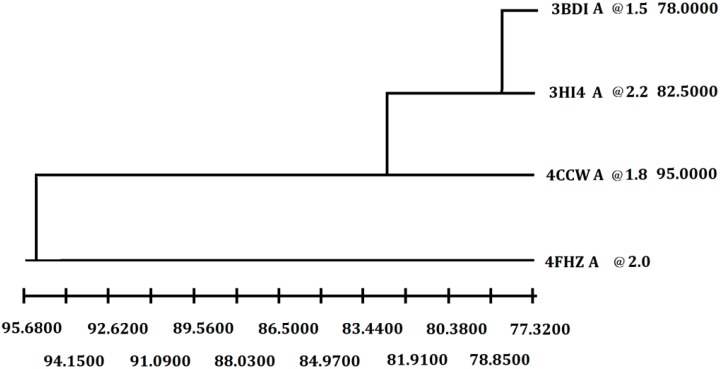
Selection of the best crystal structure template for homology modeling. Weighted pair-group average clustering based on a distance matrix. The last column represents the distance between this protein’s cluster and those below it.

In order to get the 3D model of P186_1588, the crystal structure of the carboxylesterase (PDB ID: 3BDI) from *T. acidophilum* was finally selected as the best template for the homology modeling according to the crystallographic resolution and overall sequence identity (Figure 2). In general, proteins with 30%–50% sequence identity share at least 80% of their structures [28]. The P186_1588 shares 30% of sequence identity (coverage 99%) with the selected template. After 100 models calculated by Modeller, the best P186_1588 model was selected with the lowest value of discrete optimized protein energy (DOPE) assessment score [29]. Furthermore, the geometry analysis of the model using online PROCHECK showed that 89.4% of the residues in the most favored regions of the Ramachandran plot, 10.6% of the residues in the allowed regions, and none of residues in disallowed regions (Figure S1-A). Moreover, the ProSA Z score (−7.60) for the model is also in the range of scores typically found in the proteins with similar sequence length (Figure S1-B) [30]. All of these results indicated that the model of P186_1588 was reasonable and acceptable.

**Figure 3:**
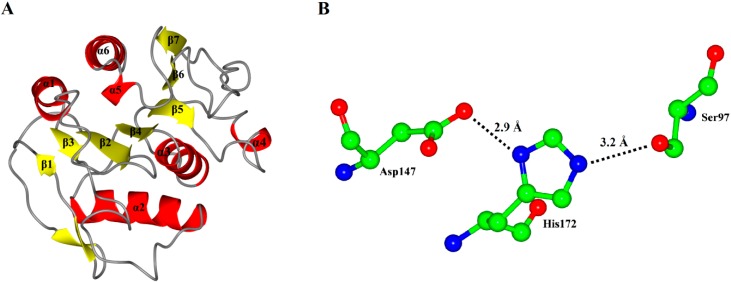
(**A**) Overall representation of P186_1588. The α-helices and β-sheets domain are in red and yellow, respectively; (**B**) The catalytic triad (Ser_97_, Asp_147_ and His_172_) of P186_1588 shown as ball-and-stick mode. The lines and numbers represent the distances of Oγ-Ser_97_/Nε-His_172_ and Nδ-His_172_/Oδ-Asp_147_ in angstroms.

As expected, the model of P186_1588 showed a typical α/β hydrolase fold with β-sheets surrounded by α-helices (Figure 3A). It contained six α-helices and seven β-sheets. The CASTp [31] program predicted that its active site (pocket) consisted of residues Gly29, Trp30, Ser31, Phe32, Pro96, Gly120, Val149, Ala173 and Tyr175. The internal part of the pocket exhibited a hydrophobic region including the residues Trp30, Phe32, Pro96, Val149, and Tyr175. The catalytic triad Ser_97_, Asp_147_ and His_172_ were located on the loops between β4-α3, β6-β7 and β7-α5, respectively. In order to complete the catalytic triad, the hydroxyl (Oγ-Ser_97_) of Ser_97_ can form a hydrogen bond (3.2 Å) with the N atom (Nε-His_172_) of His_172_ whose other N atom (Nδ-His_172_) can form a hydrogen bond (2.9 Å) with the hydroxyl (Oδ-Asp_147_) of Asp_147_ (Figure 3B).

### 2.2. Purification and Identification of the Recombinant Esterase

**Figure 4:**
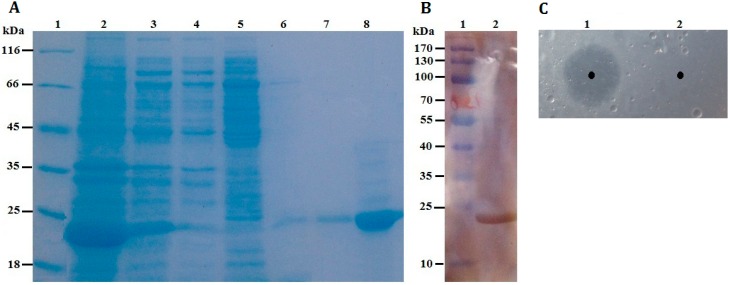
(**A**) SDS-PAGE. Lane 1: protein molecular weight markers; Lane 2: the soluble fraction of the cell lysate after induction; Lane 3: the insoluble fraction of the cell lysate after induction; Lane 4: fluid through Ni-NTA column; Lane 5: purified fraction from Ni-NTA eluted by washing buffer containing 30 mM imidazole; Lane 6: purified fraction from Ni-NTA eluted by washing buffer containing 60 mM imidazole; Lane 7: purified fraction from Ni-NTA eluted by washing buffer containing 100 mM imidazole; Lane 8: purified rP186_1588 eluted by washing buffer containing 200 mM imidazole; (**B**) Western blotting analysis. Lane 1: protein markers; Lane 2: purified rP186_1588; (**C**) Activity testing on tributyrin plate. Lane 1: rP186_1588; Lane 2: buffer solution (NTA-0).

**Figure 5:**
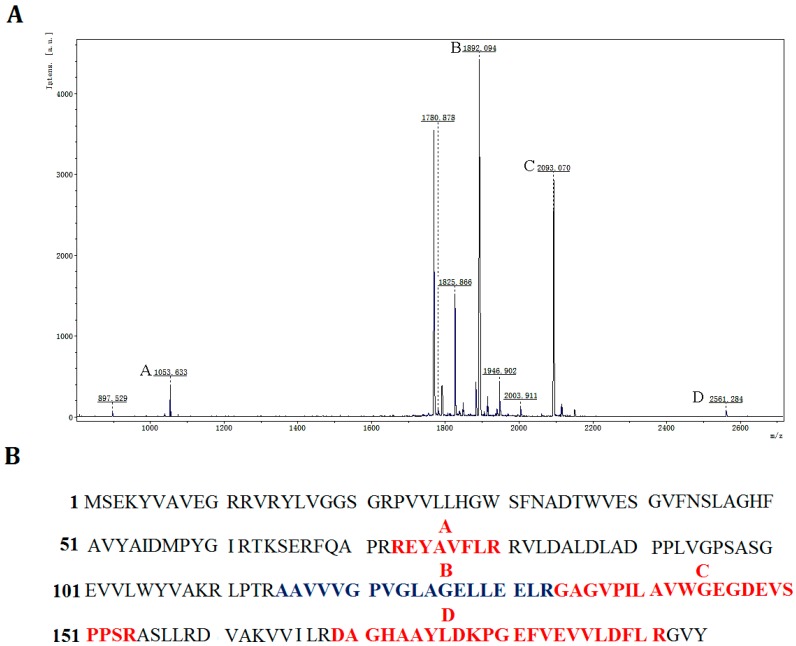
Identification of rP186_1588 by peptide mass fingerprinting analysis. (**A**) MALDI-TOF spectra of rP186_1588, *x*-axis: *m*/*z* ratio; *y*-axis: species abundance in terms of percent signal intensity. Major peaks corresponding to the predicted peptides are marked as A, B, C, and D; (**B**) The peptide fragments of rP186_1588 whose molecular weights correspond to those of the peaks shown in the peptide mass fingerprint.

In order to analyze whether the *P186_1588* expression product has an esterase activity, the corresponding protein (rP186_1588) was purified from recombinant *E. coli* BL21 (DE3). The purification parameters of rP186_1588 are summarized in Table 1. The results revealed that the purification procedure had a recovery of 33.88% in total enzyme activity and a 21.34-fold increase in enzyme specific activity. Furthermore, as shown in Figure 4A, rP186_1588 was partially soluble expressed in *E. coli*, and was found to be electrophoretically pure by SDS-PAGE with right molecular weight of approximately 23 kDa. By western blotting analysis using anti-His antibody, the purified rP186_1588 presented as a single band (Figure 4B). In order to further determine that the purified protein was the product of gene* p186_1588*, peptide mass fingerprinting was used to analyze the digested peptide fragments of rP186_1588. Four peptide fragments matched the deduced amino acid sequence of *p186_1588* (Figure 5). These results proved that the purified rP186_1588 was encoded by *p186_1588*. In order to determine whether the purified rP186_1588 was active, rP186_1588 was spotted on a tributyrin agar plate. After incubation for 12 h, a clear hydrolysis zone was formed on plate (Figure 4C), which further confirmed that rP186_1588 was functionally expressed in *E. coli* and displayed activity towards tributyrin.

**Table 1:** Purification of rP186_1588 from *E. coli* BL21 (DE3) *.

	Concentration (mg/mL)	Volume (mL)	Activity (U/mL)	Specific Activity (U/mg)	Purification Fold	Recovery (%)
Cell lysate	4.40	30.00	7.01	1.59	1	100
rP186_1588	0.28	7.50	9.50	33.93	21.34	33.88

* Esterase activity was determined with *p*-nitrophenyl acetate as substrate.

### 2.3. Biochemical Characterization of rP186_1588

**Figure 6:**
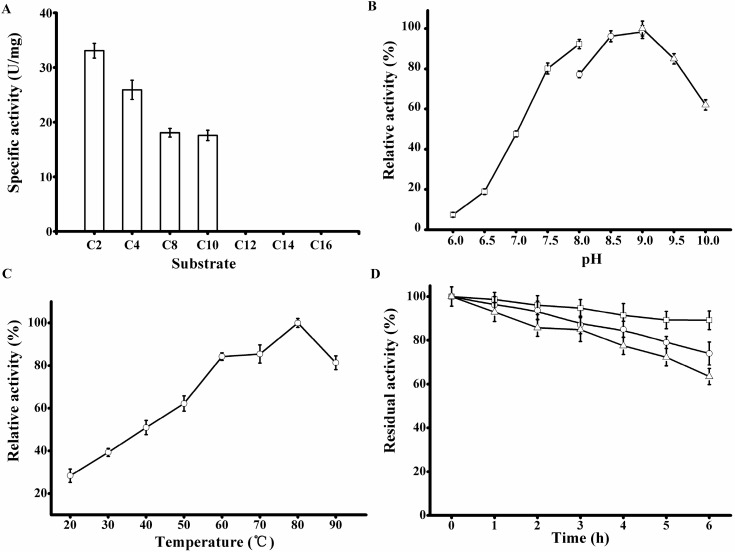
Biochemical characterization of the purified rP186_1588. (**A**) Activities of rP186_1588 towards *p*-NP esters with various chain lengths (C2, acetate; C4, butyrate; C8, caprylate; C10, decanoate; C12, laurate; C14, myristate; and C16, palmitate); (**B**) Effect of pH on the activity of rP186_1588; (**C**) Effect of temperature on the activity of rP186_1588; (**D**) Effect of temperature on stability of rP186_1588 at 70 °C (□), 80 °C (○) and 90 °C (Δ). The values represent the means of three independent experiments (Mean ± standard error).

The substrate specificity of rP186_1588 was tested using various *p*-NP esters with different acyl chain lengths (Table 2). rP186_1588 was able to hydrolyze *p*-NP esters with acyl chain length up to ten carbon atoms (Figure 6A) with the specific activity towards *p*-NPC2, *p-*NPC4,* p*-NPC8, and *p*-NPC10 of 33.07, 25.90, 18.05, and 17.57 U/mg, respectively (Table 2). Based on the substrate preference of rP186_1588, it can be concluded that it is not a lipase but an esterase. The substrate profile of rP186_1588 was different from those of other reported archaeal esterases. The carboxylesterase from* S. solfataricus* showed hydrolytic activity towards all kinds of *p*-NP esters with *p*-NPC8 as its best substrate [32]. The esterase Est from *P. calidifontis*, a member from the same genus *Pyrobaculum*, showed hydrolytic activity towards short to medium length esters with *p*-NPC6 as its best substrate [33]. In addition, the *K*_m_ values of rP186_1588 increased with the elevated chain length from *p*-NPC2 to* p*-NPC10, while the catalytic efficiency (*k*_cat_/*K*_m_) values decreased gradually (Table 2). The purified rP186_1588 displayed highest specific activity towards *p*-NPC2 with the lowest *K*_m_ value of 0.35 mM and the highest *k*_cat_/*K*_m_ value of 33.29 s^−1^·mM^−1^.

**Table 2:** Kinetic parameters for P186_1588 with *p*-NP esters.

Substrate	*K*_m_ (mM)	*k*_cat_ (s^−1^)	*k*_cat_/*K*_m_ (s^−1^·mM^−1^)	Specific Activity (U/mg)
*p*-NP acetate (C2)	0.35 ± 0.01	11.65 ± 0.48	33.29 ± 1.37	33.07 ± 1.36
*p*-NP butyrate (C4)	1.27 ± 0.04	9.12 ± 0.62	7.18 ± 0.19	25.90 ± 1.76
*p*-NP caprylate (C8)	2.64 ± 0.14	6.36 ± 0.28	2.41 ± 0.11	18.05 ± 0.79
*p*-NP decanoate (C10)	4.99 ± 0.21	6.19 ± 0.33	1.24 ± 0.07	17.57 ± 0.94

* Values are means ±SE from three independent experiments.

The purified rP186_1588 displayed the maximal activity at pH 9.0 (Figure 6B) and 80 °C (Figure 6C). These were also different from those of other reported archaeal esterases. The esterase from* S. solfataricus* exhibited maximal activity at pH of 8.0 and temperature of 85 °C [32], while the Est from *P. calidifontis* showed optimum pH and temperature at 7.0 and 90 °C respectively [33]. The thermal stability of rP186_1588 was also tested at three different temperatures (70, 80, and 90 °C). As shown in Figure 6D, rP186_1588 retained more than 70% of its original activity after 6 h of incubation at 90 °C. As for the thermal stability, the esterase from* S. solfataricus* remained about 40% of its original activity after incubation at 80 °C for 2 h [32], while the esterase Est from *P. calidifontis* showed no decrease in activity after 2 h treatment at 100 °C [33]. These results revealed that rP186_1588 possessed good thermostability.

### 2.4. Effects of Organic Solvents, Metal Ions, EDTA and PMSF on rP186_1588

The esterase activity is commonly affected by many factors, such as organic solvents, surfactants, and metal ions [2]. The maintenance of activity in organic solvents is important for industrial enzymes [2]. As shown in Table 3, in the presence of organic solvents (15%, *v*/*v*), most of the tested organic solvents including acetonitrile, acetone, methanol, ethanol, and isopropanol resulted in 21%–76% increment of the activity of rP186_1588. However, when the organic solvents were used at high concentration (30%, *v*/*v*), only methanol and acetone could still increase the activity of rP186_1588, while the other polar organic solvents decreased 20%–40% of the activity of rP186_1588. The esterase Est from* P. calidifontis* maintained 105% of residual activity after treatment with 50% of methanol for 1 h [33]. The esterase from* S. solfataricus* kept 78% of its original activity after incubation in 5% of methanol at 30 °C for 1h. However, when the temperature increased to 70 °C, only 27% of its original activity retained [32]. Comparing with these two esterases, the thermal stable rP186_1588 has good resistance to organic solvents, which also indicates that the thermostability of the enzyme correlates with its resistance to organic solvents [34]. The influence of a variety of detergents on rP186_1588 was also shown in Table 3. The detergents CTAB and Triton X-100 at low concentration (0.1%, *v*/*v*) could strongly increase the activity of rP186_1588, while the detergents Tween-20 and SDS showed inhibitory effect on the activity of rP186_1588. When the detergents were used at high concentration (1%, *v*/*v*), only the detergent CTAB could still increase the activity of rP186_1588, while the other detergents decreased 30%–60% of the activity of rP186_1588. This activity loss might be due to the 3D conformational changes of rP186_1588 caused by them [35]. This feature is different from lipases which are often activated by surfactants through increasing the access of substrates to the active center associated with the hydrophobic binding [36].

When the metal ions tested with 1 mM, Na^+^, K^+^, Ca^2+^ and Mn^2+^ showed no significant influence on the enzyme activity of rP186_1588, while Mg^2+^, Cu^2+^, Zn^2+^, and Co^2+^ could slightly decrease the activity to 94.68%, 85.33%, 92.38% and 95.11% of its original activity, respectively. However, when the metal ion concentration increased to 10 mM, rP186_1588 was inhibited by Zn^2+^ (50.97%), Mn^2+^ (88.07%) and Co^2+^ (72.68%), even completely inhibited by Cu^2+^ (4.67%). The chelating agent EDTA showed little inhibition on rP186_1588, which suggested that rP186_1588 was not a metalloenzyme and metal ions might be unnecessary for its enzymatic activity. Moreover, rP186_1588 was partially inhibited by a low concentration (1 mM) of PMSF, while it was completely inhibited by high concentration (10 mM) of PMSF. PMSF is a typical serine inhibitor and can covalently link the active serine residue. In general, PMSF always undergoes spontaneous hydrolysis at high temperature [37]. Therefore, when a low concentration of PMSF was used at high temperature, most of PMSF hydrolyzed, which resulted in little inhibitory effect on the activity of rP186_1588. However, when a high concentration was used, there was enough PMSF remaining to fully inhibit the activity of rP186_1588. These results further confirmed that rP186_1588 was a serine esterase.

**Table 3:** Effects of organic solvents, surfactants, metal ions, and inhibitors on the activity of rP186_1588.

Additives	Relative Activity(%) + SE	
Control	100	100
Organic solvents	15% (*v/v*)	30% (*v/v*)
Acetonitrile	176.67 ± 3.71	68.47 ± 0.68
Acetone	121.38 ± 2.06	122.89 ± 5.53
DMSO	84.88 ± 0.31	77.75 ± 1.48
Methanol	144.49 ± 1.01	168.47 ± 5.90
Ethanol	165.23 ± 2.31	80.13 ± 1.24
Isopropanol	143.20 ± 2.29	79.27 ± 0.60
Surfactants	0.1% (*v/v*)	1% (*v/v*)
CTAB	142.55 ± 3.79	179.91 ± 2.91
Tween-20	84.02 ± 1.48	66.52 ± 0.73
Triton X-100	130.45 ± 3.65	69.11 ± 0.29
SDS	83.15 ± 1.33	42.33 ± 0.29
Metal ions	1 mM	10 mM
Na^+^	105.32 ± 1.79	109.20 ± 1.93
K^+^	112.65 ± 2.37	117.25 ± 5.04
Ca^2+^	108.99 ± 2.47	107.12 ± 0.48
Mg^2+^	94.68 ± 2.25	119.12 ± 0.73
Mn^2+^	103.74 ± 2.80	88.07 ± 1.14
Cu^2+^	85.33 ± 2.09	4.67 ± 0.02
Zn^2+^	92.38 ± 1.57	50.97 ± 1.68
Co^2+^	95.11 ± 4.09	72.68 ± 1.96
Inhibitor	1 mM	10 mM
EDTA	96.83 ± 0.17	120.73 ± 3.75
PMSF	71.64 ± 0.70	ND *

* ND: not detected; Values are means of three replicates ± SE.

### 2.5. CD Analysis

**Figure 7:**
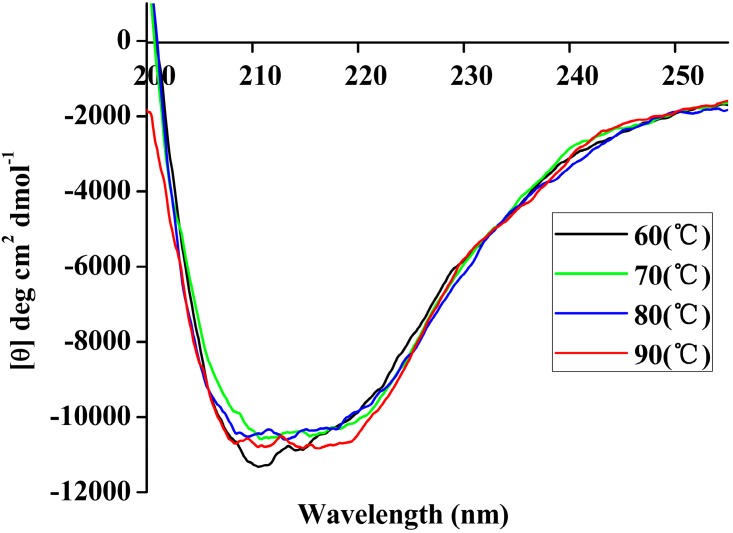
Far-UV CD spectra of rP186_1588 recorded in the 250 nm–200 nm range at different temperatures (60 °C, black; 70 °C, green; 80 °C, blue; 90 °C, red).

In order to further confirm its thermal stability, CD spectra were conducted to monitor the protein conformational change which was remarkably sensitive to far-UV [38]. As shown in Figure 7, far-UV CD spectra for rP186_1588 displayed strong negative bands in the region of 200 to 250 nm. On increasing the temperature from 60 to 90 °C, no significant structural changes were observed. These results clearly indicated that rP186_1588 maintained well at high temperatures.

### 2.6. Molecular Dynamics Simulations

**Figure 8:**
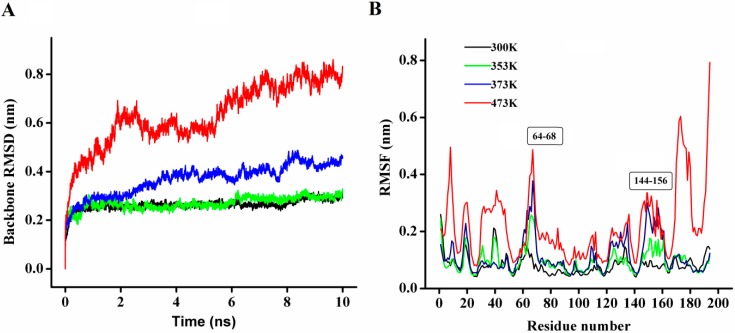
(**A**) The rmsd values for the backbone atoms of P186_1588 during 10 ns simulation at different temperatures; (**B**) The rmsf values of the residues in P186_1588 at different temperatures. The color-coding scheme is as follows: 300 K (black), 353 K (green), 373 K (blue) and 473 K (red).

In order to explore the thermostability of P186_1588, comparative MD simulations were carried out at four different temperatures (300, 353, 373, and 473 K). The dynamic behavior of the enzyme was examined by analyzing the MD simulation trajectories. As shown in Figure 8A, at 300 and 353 K simulations, the backbone RMSD (root mean square deviation) equilibrated after about 600 ps, and remained stable until the end of the simulation with RMSD value converged to 0.263 and 0.270 nm respectively. These results indicated that the global 3D structure of P186_1588 was well maintained and the protein was stable throughout the simulation. In the trajectory run at 373 K, the backbone RMSD of P186_1588 reached equilibrium after about 600 ps and maintained well to about 2.5 ns. After that, the backbone RMSD increased and attained a high value of 0.367 nm. At 473 K simulation, the backbone RMSD increased from the beginning of the simulation and reached a high value of 0.633 nm. These results indicated that increasing temperature up to 353 K showed very little effect on the stability of its native structure, and when the temperature was higher than 373 K, proteins started to unfold.

The average RMSF (root mean square fluctuations) values in the MD simulation reflect the relative flexibility of each residue in a protein—a higher rmsf value indicates less stability [20,21,39]. As shown in Figure 8B, most regions in P186_1588 showed minor fluctuation with the temperature increased from 300 to 373 K. These results revealed that most regions in P186_1588 were relatively thermal stable with certain rigidity. As reported in many studies, thermophilic enzymes are more rigid than mesophilic enzymes [40,41]. Two flexible regions including residues 64–68 (designated as region A1) and residues 144–156 (designated as region A2) showed major fluctuation, which indicated that they were thermal sensitive areas. These flexible regions (A1 and A2) are two loops connecting β3 and α2, and β6 and β7, respectively. Considering the RMSFs of the three catalytic residues, Asp_147_ was the largest fluctuation residue that was located in the flexible region A2, while the RMSFs of Ser_97_ and His_172_ were relative low at high temperatures. Therefore, the flexibility of region A2 might favor the entry of the substrates and/or catalysis [42]. However, the region A1 is far away from the catalytic center in spatial structure (Figure S2), so, this area would be regarded as the hotspot for protein engineering in order to further improve its thermostability [43].

The radius of gyration (Rg) parameter, calculated as the RMS distance of atoms from the protein’s center, reflects the compactness of protein structure. In general, reduced Rg (higher compactness) has been identified as a protein stabilizing factor [10]. As shown in Table 4, P186_1588 had the most compact structure at the lowest temperature (300 K, Rg: 1.597 nm). Moreover, P186_1588 displayed similar compactness at 353 K (Rg: 1.617 nm) and 373 K (Rg: 1.622 nm) which were a little less compact than it at 300 K. However, the average Rg value increased to 1.672 nm at the temperature of 473 K. These results also indicated that P186_1588 had good thermal stability at temperatures below 373 K.

**Table 4:** Average of Rg, SASA and average number of protein-protein and protein-solvent H-bonds in P186_1588 at different temperature simulations.

P186_1588	300 K	353 K	373 K	473 K
Backbone RMSD (nm)	0.263	0.270	0.367	0.633
Rg (nm)	1.597	1.617	1.622	1.672
SASA (nm^2^)	105.926	111.506	112.778	119.897
PSASA (nm^2^)	54.597	55.215	57.428	56.007
ASASA (nm^2^)	51.329	56.291	55.349	63.891
Protein-protein H-bond	113	109	104	96
Protein-solvent H-bond	403	390	383	303

Rg, SASA and H-bonds denote radius of gyration, solvent accessible surface area and hydrogen bonds respectively. PSASA and ASASA denote polar and apolar solvent accessible surface area respectively.

The solvent accessible surface area (SASA) reflects the exposure of protein atoms to solvent, which can be obtained by calculating the surface area of atom in contact with solvent molecules [22]. In our study, the total SASA values showed a slight increase with the temperature rising from 300 to 473 K. This profile is in agreement with the variation trend of Rg values. These results revealed that P186_1588 became less compact with more solvent penetration into the internal core of the protein at high temperatures. Comparing the variation of hydrophilic SASA (PSASA) and hydrophobic SASA (ASASA), the increase of total SASA is mostly attributed to the hydrophobic residues (Table 4). In general, the non-polar side chains of protein always cluster together into the interior through the hydrophobic interactions [21]. The increase in the hydrophobic SASA indicated that more non-polar side chains became to be exposed to the solvents at high temperatures. The average number of intramolecular hydrogen bonds was also calculated at different temperature simulations. As shown in Table 4, with the increase in temperature, there was a slight decrease in number of the hydrogen bond. However, average numbers of protein-solvent interactions decreased more sharply at high temperature (473 K) (Table 4).

To gain more insight into the effect of temperature on the conformation of P186_1588, the second structure of the protein was also calculated according to the DSSP algorithm. As shown in Table 5, the content of α-helix and β-sheet in P186_1588 maintained well in the simulation period at temperature of 300, 353, and 373 K. However, the content of α-helix and β-sheet reduced sharply at temperature of 473K, which indicated that α-helix and β-sheet were gradually disordered at high temperature. These resulted revealed that P186_1588 started to unfold at temperature higher than 373K, which corresponded well with the RMSd results and CD results.

**Table 5:** Average second structure contents in P186_1588 trajectories obtained at different temperatures.

P186_1588	300 K	353 K	373 K	473 K
Coil	0.22	0.26	0.23	0.28
β-Sheet	0.21	0.20	0.22	0.17
β-Bridge	0.00	0.01	0.01	0.01
Bend	0.15	0.16	0.18	0.22
Turn	0.16	0.14	0.11	0.16
α-Helix	0.21	0.22	0.24	0.10
3_10_-Helix	0.05	0.03	0.02	0.05

**Figure 9:**
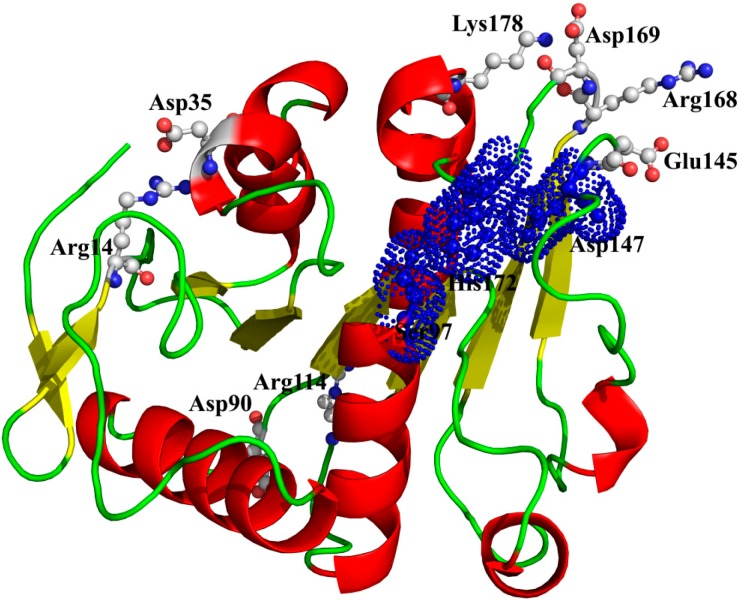
The spatial distribution of the important salt bridges in P186_1588. The backbone of the protein is represented in cartoon. Residues forming the ion pairs are represented in ball-and-stick mode. The catalytic residues (Ser_97_, Glu_147_ and His_172_) were shown as dot.

Electrostatic interactions of ion pairs in thermophilic enzyme may contribute to its stability at high temperature [6,21]. Molecular dynamic simulations can provide valuable information for identifying the critical ion pairs, and also help us to design more favorable interaction for mesophilic proteins by introducing new charged residues [44]. From the amino acid component of P186_1588, we found that it contained 45 charged amino acid residues including Lys, Arg, Asp, and Glu. Many ion pairs that formed by the positively charged and negatively charged residues have been found during the simulations at 300, 353, 373, and 473 K (Data not shown). In general, salt bridges became weaken and their occupancy times decreased with the temperature increasing. However, among these ion pairs, four ion pairs (Arg^14^-Asp^35^, Arg^114^-Asp^9^°, Lys^178^-Asp^169^, and Arg^168^-Glu^145^) could still maintain well even at high temperatures, suggesting a crucial role in stabilizing this enzyme (Table 6) [42]. As shown in Figure 9, two ion pairs (Arg^14^-Asp^35^ and Arg^114^-Asp^9^°) are far away from the catalytic center, while the other two ion pairs (Lys^178^-Asp^169^ and Arg^168^-Glu^145^) are near to the catalytic center. Especially, in the ion pair of Arg^168^-Glu^145^, the residue Arg_168_, near to the catalytic residue His_172_, is located on a loop structure, and the other residue Glu_145_, near to the catalytic residue Asp_147_, is located on another loop structure. Therefore, this ion pair (Arg^168^-Glu^145^) may have an important role in maintaining the distance between the catalytic residues Asp_147_ and His_172_.

**Table 6:** Important salt bridge interactions of P186_1588 at 300K, 353K, 373K, and 473K simulation and their occupation time *.

Salt Bridge	300 K		353 K		373 K		473 K	
	Occupancy Time (%)	Distance (nm)	Occupancy Time (%)	Distance (nm)	Occupancy Time (%)	Distance (nm)	Occupancy Time (%)	Distance (nm)
R14-D35	100	0.175	99	0.185	97	0.228	85	0.298
R114-D90	100	0.288	80	0.441	75	0.402	54	0.495
K178-D169	99	0.308	99	0.307	99	0.306	84	0.416
R168-E145	99	0.332	99	0.370	78	0.402	71	0.466

* The average distance of MD simulation. Only ion-pairs with a distance less than 0.5 nm are reported. Occupancy time is the percentage of structures where the salt bridge distance is less than 0.5 nm. Only ion-pairs with time occupancy more than 50% are reported.

### 2.7. Docking Analysis and Binding Free Energy Decomposition

**Figure 10:**
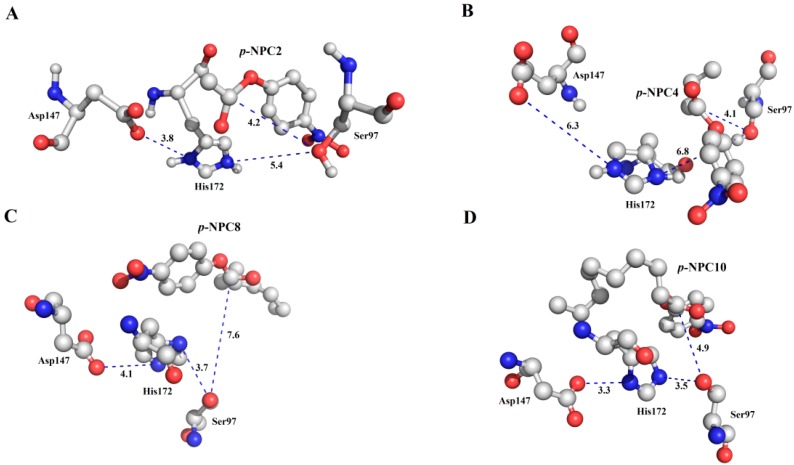
The binding mode of the complexes between P186_1588 and *p*-NP esters. (**A**) The binding pose for the complex of P186_1588 and *p*-NPC2; (**B**) The binding pose for the complex of P186_1588 and *p*-NPC4; (**C**) The binding pose for the complex of P186_1588 and *p*-NPC8; (**D**) The binding pose for the complex of P186_1588 and *p*-NPC10.

**Figure 11:**
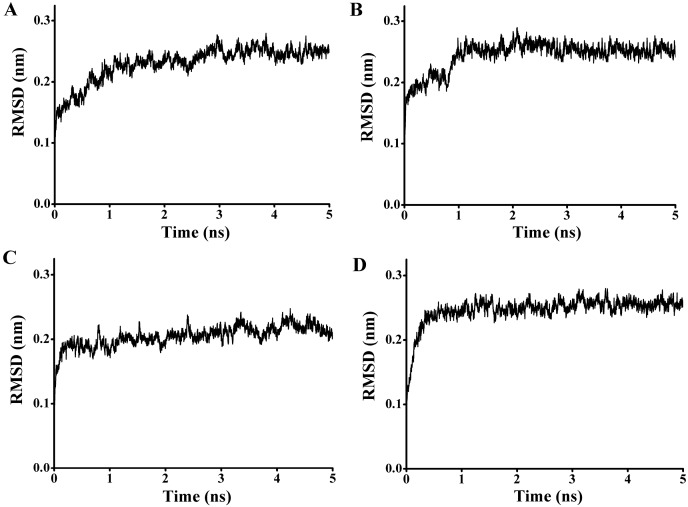
The rmsd values for the backbone atoms of the docked complex during 5 ns simulations. (**A**) The rmsd values for the complex of P186_1588 and *p*-NPC2; (**B**) The rmsd values for the complex of P186_1588 and *p*-NPC4; (**C**) The rmsd values for the complex of P186_1588 and *p*-NPC8; (**D**) The rmsd values for the complex of P186_1588 and *p*-NPC10.

As characterized in the Section 2.3, rP186_1588 showed ability to hydrolyze short to medium chain length *p*-NP esters. In order to explain its substrate specificity, four substrates (*p*-NPC2, *p*-NPC4, *p*-NPC8 and *p*-NPC10) were docked into P186_1588. In the Figure 10, all the substrates were located in a reasonable orientation with the distance between hydroxyl (Oγ-Ser_97_) and the carbonyl carbon of the substrate from 4.1 to 7.6 Å. To get a reliable binding free energy, all the complexes were performed for MD simulations. After 5 ns MD simulation, RMSD values of all the complexes were stable after approximately 3000 ps, indicating that the simulation systems have equilibrated (Figure 11). The equilibrated systems were then used to calculate the binding free energy for each enzyme-substrate complex using MM-PBSA approach. As shown in Table 7, negative binding free energy values (Δ*G*_bind__ing_) revealed that the binding between the enzyme and the *p*-NP esters were spontaneous. However, the Δ*G*_bind__ing_ value rose with the increase of the carbon chain length of substrate. The Δ*G*_bind__ing_ between P186_1588 and *p*-NPC2 showed the lowest binding free energy (−39.54 kcal·mol^−^^1^) in the four enzyme-substrate complexes. These results predicted that P186_1588 would show the highest affinity towards *p*-NPC2. This profile is in harmony with the kinetic experiment that rP186_1588 displayed the lowest *K*_m_ value towards *p*-NPC2 (Table 2).

To get insight into the driving force for the binding interaction, the binding free energy items were also analyzed. The binding free energy component in Table 7 shows that the van der Waals interactions (Δ*E*_vdW_) are much larger than other energy components, which reveals that the van der Waals interactions are the major driving force for the binding between P186_1588 and *p*-NP esters. In addition, the electrostatic energy (Δ*E*_ele_) shows as negative values, while the polar contribution to the solvation free energy (Δ*E*_PB_) displays as positive values, which indicates that the electrostatic interactions would be offset by the unfavorable electrostatic solvation free energy. This result is expected since the electrostatic interaction is generally anti-correlated with the electrostatic solvation free energy [45].

**Table 7:** Binding free energy (in kcal·mol^−^^1^) for *p*-NP esters binding to P186_15588 *.

	Δ*E*_ele_	Δ*E*_vdW_	Δ*E*_PB_	Δ*E*_SA_	Δ*G*_gas_	Δ*G*_sol_	Δ*G*_bind__ing_
***p*-NPC2**	−3.31(0.32)	−42.84(0.33)	10.35(0.25)	−3.74(0.02)	−46.15(0.40)	6.61(0.26)	−39.54(0.31)
***p*-NPC4**	−0.00(0.00)	−37.46(0.28)	6.25(0.08)	−4.28(0.01)	−37.46(0.28)	1.97(0.08)	−35.49(0.27)
***p*-NPC8**	−2.22(0.14)	−29.58(0.23)	6.80(0.11)	−3.26(0.02)	−31.80(0.29)	3.54(0.10)	−28.26(0.24)
***p*-NPC10**	−0.32(0.02)	−30.72(0.34)	7.79(0.23)	−3.84(0.03)	−31.04(0.42)	3.85(0.23)	−27.18(0.34)

* **Δ***G*_gas_ = **Δ***E*_ele_+ *E*_vdW_; **Δ***G*_sol_ = **Δ***E*_PB_ + **Δ***E*_SA_; **Δ***G*_bind__ing_ = **Δ***G*_gas_ + **Δ***G*_sol_.

**Figure 12:**
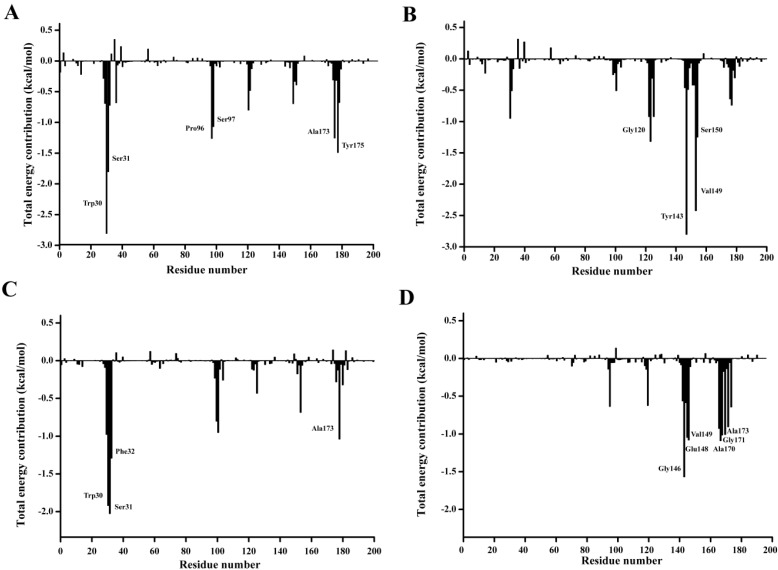
Residue-residue interaction spectra for all kinds of complexes according to the MM-PBSA method. The x-axis denotes the residue number, and the y-axis denotes the interaction energy between the P186_1588 and each residue. The important residues (<−1 kcal·mol^−1^) for binding are marked by corresponding texts. (**A**) The residue-residue interaction spectra for the complex of P186_1588 and *p*-NPC2; (**B**) The residue-residue interaction spectra for the complex of P186_1588 and *p*-NPC4; (**C**) The residue-residue interaction spectra for the complex of P186_1588 and *p*-NPC8; (**D**) The residue-residue interaction spectra for the complex of P186_1588 and *p*-NPC10.

In order to qualitatively obtain more details of the residue energy contribution that played an important role in the *p-*NP ester binding, the binding free energy was decomposed on a per-residue level with MM-PBSA method. The per residue contribution profile are shown in Figure 12. Six residues including Trp30, Ser31, Pro96, Ser97, Ala173, and Tyr175 are observed to significantly contribute to the substrate binding of *p*-NPC2. Four residues Gly120, Trp143, Val149, and Ser150 have major contribution to the binding of *p*-NPC4. As for the binding of *p*-NPC8, Trp30, Ser31, Phe32, and Ala175 are found to make remarkably contribution. As for the binding of *p*-NPC10, Gly146, Glu148, Val149, Ala170, Gly171, and Ala173 are shown important contribution. In addition, as shown in Table 8, the van der Waals energy contribution of each residue are much larger than the corresponding electrostatic energy contribution. These results reveal that these residues contribute their substrate affinity mainly through van der Waals interactions. Moreover, the total interaction energy for *p*-NPC2 (−9.69) and *p*-NPC4 (−7.63) were lower than those for *p*-NPC8 (−6.28) and *p*-NPC10 (−6.80). These results revealed that the binding pocket could provide a better hydrophobic environment for short chain *p*-NP esters. In the complex of P186_1588 and *p*-NPC2, and P186_1588 and *p*-NPC8, Trp30 provided the strongest van der Waal energy, which suggested that it played a crucial role in the binding of *p*-NPC2 and *p*-NPC8. These results revealed that improving the hydrophobic environment through protein engineering would further enhance its activity towards short chain *p*-NP esters.

**Table 8:** The total energy (*E*_total_), van der Waal energy (*E*_vdW_), and electrostatic energy (*E*_ele_) between substrates and individual residues (*E*_total_ < −1.0 kcal·mol^−1^ listed in energy rank order).

*p*-NPC2	*E*_vdW_	*E*_ele_	*E*_total_	*p*-NPC4	*E*_vdW_	*E*_ele_	*E*_total_
Trp30	−2.68	−0.13	−2.81	Gly120	−1.28	−0.04	−1.32
Ser31	−1.14	−0.66	−1.80	Trp143	−2.78	−0.02	−2.80
Pro96	−1.16	−0.10	−1.26	Val149	−2.34	0.08	−2.42
Ser97	−1.01	−0.06	−1.07	Ser150	−1.21	−0.04	−1.25
Ala173	−1.39	0.13	−1.26				
Tyr175	−1.60	0.11	−1.49				
Total	−8.98	−0.71	−9.69	Total	−7.61	−0.02	−7.63
***p*-NPC8**	***E*_vdW_**	***E*_ele_**	***E*_total_**	***p*-NPC10**	***E*_vdW_**	***E*_ele_**	***E*_total_**
Trp30	−2.14	0.22	−1.92	Gly146	−1.35	−0.22	−1.57
Ser31	−1.14	−0.89	−2.03	Glu148	−1.47	0.43	−1.04
Phe32	−1.42	0.13	−1.29	Val149	−1.10	0.02	−1.08
Ala173	−0.93	−0.11	−1.04	Ala170	−1.08	−0.01	−1.09
				Gly171	−1.20	0.19	−1.01
				Ala173	−1.02	0.01	−1.01
Total	−5.63	−0.65	−6.28	Total	−7.22	0.42	−6.80

## 3. Experimental Section

### 3.1. Bacterial Strains, Plasmids, Enzymes and Chemicals

*E. coli* BL21 (DE3) and vector pET-28a (+) (Invitrogen, Carlsbad, CA, USA) were used for heterologous expression of the esterase. *E. coli* was cultivated at 37 °C in Luria-Baertani (LB) broth or on agar plates. The plasmid extraction kit and DNA purification kit were purchased from Omega (USA). Taq DNA polymerase, T4 DNA ligase, restriction endonucleases, and isopropyl-β-d-thiogalactopyranoside (IPTG) were bought from TaKaRa (Dalian, China). To validate the accuracy of our gene insertion, DNA sequencing was performed at the Shanghai Sunny Biotechnology Company (Shanghai, China).

Substrates including *p*-nitrophenyl-acetate (*p*-NPC2), *p*-nitrophenyl-butyrate (*p*-NPC4), *p*-nitrophenyl-caproate (*p*-NPC8), *p*-nitrophenyl-decanoate (*p*-NPC10), *p*-nitrophenyl-laurate (*p*-NPC12), *p*-nitrophenyl-myristate (*p*-NPC14), *p*-nitrophenyl-palmitate (*p*-NPC16) were purchased from Sigma-Aldrich (St. Louis, MO, USA). All other reagents used were of analytical grade and commercially available from Sinopharm Chemical Reagent Co., Ltd., Shanghai, China.

### 3.2. Expression and Purification of rP186_1588

The putative carboxylesterase gene *P186_1588* (accession number: G7VG08) from *Pyrobaculum* sp. 1860 was synthesized by the Shanghai Sunny Biotechnology Company. Then, the gene *P186_1588* was double digested with *Nde* I*/Bam* HI and ligated into the plasmid pET-28a (+). After verification of the gene insertion by sequencing, the recombinant plasmid (pET*-1588*) was transformed into *E. coli* BL21 (DE3) competent cells.

The transformant was grown aerobically at 37 °C in LB broth containing 50 μg/mL kanamycin. When the culture density reached 0.5 at OD_600_, cells were induced by IPTG (isopropyl-d-1-thiogalactopyranoside) at a final concentration of 0.4 mM. After induction at 20 °C for 18 h, the cells were harvested by centrifugation at 12,000 rpm for 10 min. Cell pellet was then resuspended in lysis buffer (20 mM Tris-HCl, 0.5 M NaCl, pH 8.0) and disrupted by ultrasonic treatment for 10 min in 5 s on/5 s off cycles. The cell lysate was centrifuged at 12,000 rpm for 30 min at 4 °C, and the supernatant was applied on the Ni-NTA affinity chromatography column (GE healthcare, Uppsala, Sweden) to purify the recombinant esterase using an imidazole concentration gradient (0, 30, 60, 100, and 200 mM) in washing buffer (20 mM Tris-HCl, 0.5 M NaCl, pH 8.0). Finally, the purified enzyme (rP186_1588) was dialyzed to remove the imidazole. Protein concentration was determined by the Bradford method using bovine serum albumin (BSA) as standard [46].

### 3.3. SDS-PAGE, Western Blot, MS Analysis and Activity Testing

Sodium dodecyl sulfate-polyacrylamide gel electrophoresis (SDS-PAGE) was performed according to the method reported by Laemmli [47]. The separated proteins were visualized through staining with Coomassie brilliant blue R-250. The single protein band after purification was confirmed by peptide mass fingerprinting [48]. In addition, after SDS-PAGE, the proteins were transferred onto polyvinylidene fluoride (PVDF) membrane. The membrane was blocked with 5% milk, followed by incubation with anti-His tag (1:1000) primary antibody, and then with horseradish peroxidase (HRP)-conjugated goat anti-rabbit IgG secondary antibody. Finally, the blot was visualized by HRP-DAB kit (Tiangen, Beijing, China) [49]. In order to determine whether the recombinant enzyme was active, rP186_1588 was spotted on the tributyrin plate (water, tributyrin (1%, *v*/*v*), agar (1.5%, *w*/*v*)) which was incubated at 37 °C to detect the formation of hydrolysis zone.

### 3.4. Biochemical Characterization

Esterase activity was assayed by measuring the amount of *p*-nitrophenol (*p*-NP) released from *p*-nitrophenyl ester [49]. In a standard assay, the total reaction system contains 50 mM Tris-HCl buffer (pH 8.0), 500 μM *p-*NP ester, ethanol (1%, *v*/*v*) and enzyme solution [49]. After the reaction system prewarmed at 80 °C for 5 min, the reaction was started by the addition of the purified recombinant enzyme. Following incubation at 80 °C for 5 min, the reaction was terminated by rapid cooling on ice water. The background hydrolysis of the substrate was deducted by using a blank control without the addition of the enzyme solution. One unit (U) of enzyme activity is defined as the amount of enzyme which librates 1 μmole of *p-*NP per minute [49].

The substrate specificity of rP186_1588 was measured using a variety of *p*-NP esters (*p*-NPC2, *p*-NPC4, *p*-NPC8, *p*-NPC10, *p*-NPC12, *p*-NPC14, and* p*-NPC16) under the standard assay conditions. The effect of pH on rP186_1588 was tested at 80 °C at pH ranging from 6.0 to 10.0 in various buffers: 50 mM NaH_2_PO_4_/Na_2_HPO_4_ buffer (pH 6.0–8.0), 50 mM Tris-HCl buffer (pH 8.0–9.0), and 50 mM glycine/NaOH buffer (pH 9.0–10.0) [2]. The optimum temperature for rP186_1588 was determined by assaying this enzyme at temperatures between 20 to 90 °C at pH 9.0 using *p*-NP butyrate as the substrate. For stability determination, the purified rP186_1588 was incubated in 50 mM Tris-HCl (pH 9.0) at 70, 80, and 90 °C for different periods. Subsequently, the residual activity was analyzed at its optimum temperature. The kinetic parameters of rP186_1588 were measured in 50 mM Tris-HCl (pH 9.0) at 80 °C using the substrates of *p*-nitrophenyl esters at different concentrations (0.1, 0.2, 0.3, 0.4, 0.5, 1, 2 and 3 mM) [49]. The Michaelis-Menten constant (*K*_m_) and the maximum velocity (*V*_max_) for each substrate were calculated from Lineweaver-Burk plots using the Microsoft Excel software.

In order to estimate the effects of all kinds of additives on rP186_1588, the enzyme solutions of rP186_1588 were respectively treated for 1 h with various additives including 1 or 10 mM metal ions (Na^+^, K^+^, Ca^2+^, Mg^2+^, Mn^2+^, Cu^2+^, Zn^2+^, and Co^2+^), 15% or 30% (*v*/*v*) organic solvents (acetonitrile, methanol, ethanol, isopropanol, acetone, and DMSO), 0.1% or 1% (*v*/*v*) detergents (CTAB, SDS, Tween-20, and Triton X-100), and 1 or 10 mM enzyme inhibitors (EDTA and PMSF). Then, the residual activities were determined by measuring the enzyme activity under the standard assay condition. All assays were conducted in three times independently, and the reaction mix without any additives was taken as control (100%) [2].

### 3.5. Circular Dichroism (CD) Spectroscopy

The effect of temperature on enzyme conformation was studied by circular dichroism (CD) spectroscopic technique. CD measurements were carried out on a JASCO J-810 spectropolarimeter (JASCO, Tokyo, Japan) [37]. CD spectra were recorded in 0.1 cm path length quartz cell cuvette using 0.1 mg/mL purified rP186_1588 in 100 mM sodium phosphate buffer (pH 8.0). The warm-up periods of 60 to 90 °C and wavelength scan of 200 to 250 nm were considered. The data pitch, bandwidth, response, scanning speed, and accumulation were set to be 0.1 degree, 1 nm, 2 s, 200 nm/min and 8 times, respectively. The spectra of protein samples were subtracted by blanks. Molecular ellipticity is determined as [θ]λ = [100 × (MRW) × θobs/(*c* × *l*)], where MRW is 110 for this protein, θobs is the observed ellipticity in degrees at a given wavelength, *c* is the protein concentration in mg/mL and *l* is the length of the light path in cm [48].

### 3.6. Structural Modeling and Molecular Dynamic Simulation

Protein sequence similarity searching was performed using online BLASTP searches against the protein data bank [30]. Multiple sequence alignment was carried out using Clustal W (version 1.83) and presented using ESPript 2.2 [50]. The 3D structure of the target protein was constructed by MODELLER (version 9.12) [29]. The generated structures were validated by PROCHECK [51] and evaluated by ProSA [52].

In order to explore the thermostability of P186_1588, MD simulations were done using GROMACS v. 4.6.3 utilizing the GROMOS 43a1 force field [21]. The modeled structure, used as the starting structure, was placed in a cubic simulation box with the distance between the protein outer layer and simulation box set at 10 Å. Then, 10,325 SPC water molecules were added into the system with the addition of 1 Na^+^ to neutralize the system. Initially, the system was subjected to energy minimization using steepest descent method until the maximum of force (*F*_max_) on any atom was <100 kJ·mol^−^^1^·nm^−^^1^. Then, the system was equilibrated by position restrained simulation for 200 ps each at four different temperatures including 300, 353, 373 and 473 K. Finally, 10-ns MD simulations were performed each at four different temperatures without any position constraints. The LINCS algorithm was used to constrain bond length using a time step of 1 fs for all calculations. To maintain the simulated systems at a constant temperature and pressure, the Berendsen thermostat was applied with a coupling time of 0.1 and 2.0 ps [42]. Particle-Mesh Ewald method was used to calculate the electrostatic interaction with interpolation order of 4 and a grid spacing of 0.12 [42]. The van der Waals and Coulomb interactions were treated by using a cutoff of 1.0 nm. The non-bonded pair lists were updated every 10 steps and conformations were stored every 2 fs. GROMACS analysis tools were used to analyze MD trajectories.

### 3.7. Docking and Calculation of Binding Free Energy by MM-PBSA Method

In order to study the substrate specificity of P186_1588, AutoDock (version 4.2) was used to perform docking analysis [53]. The modeled structure of P186_1588 after energy minimization was selected as receptor for docking in order to obtain a reliable binding mode [54,55]. The structures for each substrate were constructed using ChemBio3D [56]. Polar hydrogen atoms were added to both receptor and ligands. Gasteiger partial charges were calculated on protein and ligand using AutoDock Tools. Flexible torsions in the ligand were assigned to allow the acyclic dihedral angles rotated freely. A grid box of 60 × 60 × 60 Å was defined around the catalytic Ser_97_ to embrace the binding pocket for docking calculation. With all the other parameters at their default setting, docking searches of 100 conformations were executed using the Lamarckian genetic algorithm and a maximum number of 2.5 × 10^6^ energy evaluations. Finally, the binding poses of P186_1588 with *p*-NP esters were selected considering the binding energy and the catalytic mechanism of the enzyme.

The best structures for each complex were then performed MD simulations using Amber 12 packages [57]. The GAFF and AMI-BCC method were employed to set the ligand’s parameters and charges [58,59]. The Amber ff99SB force field was chosen to depict the protein parameters. The complex was solvated with TIP3P water molecules in a cubic box with 10 Å extending in each direction from the solute, and Na^+^ were added to neutralize the system. The system was initially minimized to remove bad contacts using the descent method plus conjugate gradient method. The system was then subjected to 100 ps of heating in a NVT ensemble from 0 to 300 K, and 100 ps of density equilibration with weak restraint (10 kcal/(mol-Å^2^)) on the complex followed by 500 ps of constant pressure with unrestrained equilibration at 300 K. The productive MD simulation was then performed for 5 ns at constant temperature (300 K) and constant pressure (1 atm). The SHAKE algorithm was used to constrain all covalent bonds involving hydrogen atoms. The PME method was employed to treat the long-range electrostatic interactions. The cutoff distances for nonbonded interactions were set at 12 Å.

Molecular Mechanics Poisson-Boltzmann Surface Area (MM-PBSA) in Amber 12 is a well-established method to calculate the binding free energy for the enzyme-substrate complex [57]. In MM-PBSA, the enzyme-substrate binding free energy was calculated using the following equation:

**Δ***G*_binding_ = **Δ***G*_complex_ − [**Δ***G*_protein_ + **Δ***G*_ligand_] (1)

**Δ***G* = **Δ***H* − *T***Δ***S* ≈ **Δ***E*_MM_ + **Δ***G*_sol_ − *T***Δ***S* (2)

**Δ***E*_MM_ = **Δ***E*_int_ + **Δ***E*_ele_ + **Δ***E*_vdW_ (3)

**Δ***G*_sol_ = **Δ***G*_PB_ + **Δ***G*_SA_ (4)

In Equation (1), the binding free energy (**Δ***G*_binding_) is the difference between the free energy of the complex (**Δ***G*_complex_) and the protein (**Δ***G*_protein_) as well as the ligand (**Δ***G*_ligand_). Each free energy in Equatuion (1) was calculated using Equations (2)–(4): the **Δ***E*_MM_, **Δ**G_sol_ and -*T***Δ***S* are the changes of the gas phase MM energy, the solvation free energy, and the conformational entropy upon binding, respectively. **Δ***E*_MM_ includes **Δ***E*_int_ (internal energy: bond, angle, and dihedral energies), **Δ***E*_ele_ (electrostatic), and **Δ***E*_vdW_ (van der Waals). **Δ***G*_sol_ is the sum of electrostatic solvation energy (polar contribution, **Δ***G*_PB_), and the nonelectrostatic salvation component (nonpolar contribution, **Δ***G*_SA_). The polar contribution is calculated using PB model, and the nonpolar contribution is estimated by the solvent accessible surface area using the LCPO method. Similar to many investigations, the entropy contribution (*T***Δ***S*) was neglected in this study since our aim was to analyze the relative energy contribution of amino acids residues in enzyme-substrate complex formation [45,57]. In our study, a total of 100 frames retrieved from the last 1 ns production trajectory with an interval of 10 ps were used for the binding free energy calculation.

### 3.8. Site-Directed Mutagenesis

The site-directed mutageneses at positions of the predicted catalytic triad were performed using QuickChange™ Site-directed Mutagenesis kit (Stratagen, La Jolla, CA, USA) [36]. Mutagenic primers designed by Primer X [2] included S97A-F: 5'-ATCCTCCTCTGGTGGGGCCTgcCGCCTCTGGCGA-3'; S97A-R: 5'-TCGCCAGAGGCGgcAGGCCCCACCAGAGGAGGAT-3'; D147N-F: 5'-GTCTGGGGGGAGGGGaATGAGGTGTCTCCAC-3'; D147N-R: 5'-GTGGAGACACCTCATtCCCCTCCCCCCAGAC-3'; H172L-F: 5'-GATTTTGAGAGACGCGGGGCtCGCCGCCTATCTAGACAAG-3'; and H172L-R: 5'-CTTGTCTAGATAGGCGGCGaGCCCCGCGTCTCTCAAAATC-3', respectively (modified codons were underlined). Plasmid of pET-*1588* was used as template for whole plasmid PCR. After being digested with *Dpn* I, the PCR product was transformed into *E. coli* DH5α. After sequencing, each of the three mutant plasmids was transformed into *E. coli* BL21 (DE3) for gene expression. Mutant enzymes were purified with hexahistidine tag and tested under standard conditions for their catalytic activities.

## 4. Conclusions

In conclusion, a putative carboxylesterase gene from the archaeon *Pyrobaculum* sp. 1860 was successfully expressed in *E. coli*. Biochemical characterization revealed that rP186_1588 was a thermophilic esterase with high thermal stability, and it was stable in presence of organic solvents. These desirable characteristics made it a potential candidate for industrial applications. Moreover, comparative molecular simulations at different temperatures (300, 353, 373 and 473 K) revealed that its thermostability was associated with its conformational rigidity. Four ion pairs which maintained well at four different temperatures were identified as critical ion-pairs for thermostability of P186_1588. The binding free energy analysis by MM-PBSA method revealed that the van der Waals interaction played a major role in *p*-NP ester binding for P186_1588. Our results provide insights into the molecular structures of this archaeal esterase, and may help to its further protein engineering for industrial applications.

## Supplementary Materials

Supplementary Figures can be found at http://www.mdpi.com/1422-0067/15/9/16885/s1.

## Supplementary Files

Supplementary File 1

## References

[1] Brault G., Shareck F., Hurtubise Y., Lepine F., Doucet N. (2012). Isolation and characterization of EstC, a new cold-active esterase from *Streptomyces coelicolor* A3(2). PLoS One.

[2] Shao H., Xu L., Yan Y. (2013). Isolation and characterization of a thermostable esterase from a metagenomic library. J. Ind. Microbiol. Biotechnol..

[3] Schmidt R.D., Verger R. (1998). Lipase: Interfacial enzymes with attractive applications. Angew. Chem. Int. Ed..

[4] Gupta R., Gupta N., Rathi P. (2004). Bacterial lipases: An overview of production, purification and biochemical properties. Appl. Microbiol. Biotechnol..

[5] Li X., Qian P., Wu S.G., Yu H.Y. (2014). Characterization of an organic solvent-tolerant lipase from *Idiomarina* sp. W33 and its application for biodiesel production using Jatropha oil. Extremophiles.

[6] Vieille C., Zeikus G.J. (2001). Hyperthermophilic enzymes: Sources, uses, and molecular mechanisms for thermostability. Microbiol. Mol. Biol. Rev..

[7] Van den Burg B. (2003). Extremophiles as a source for novel enzymes. Curr. Opin. Biotechnol..

[8] Atomi H., Imanaka T. (2004). Thermostable carboxylesterases from hyperthermophiles. Tetrahedron: Asymmetry.

[9] Yu S., Yu S., Han W., Wang H., Zheng B., Feng Y. (2010). A novel thermophilic lipase from *Fervidobacterium nodosum* Rt17-B1 representing a new subfamily of bacterial lipases. J. Mol. Catal. B Enzym..

[10] Noorbatcha I.A., Sultan A.M., Salleh H.M., Amid A. (2013). Understanding thermostability factors of *Aspergillus niger* PhyA phytase: A molecular dynamics study. Protein J..

[11] Palm G.J., Fernandez-Alvaro E., Bogdanovic X., Bartsch S., Sczodrok J., Singh R.K., Bottcher D., Atomi H., Bornscheuer U.T., Hinrichs W. (2011). The crystal structure of an esterase from the hyperthermophilic microorganism *Pyrobaculum calidifontis* VA1 explains its enantioselectivity. Appl. Microbiol. Biotechnol..

[12] Dong G., Vieille C., Savchenko A., Zeikus J.G. (1997). Cloning, sequencing, and expression of the gene encoding extracellular alpha-amylase from *Pyrococcus furiosus* and biochemical characterization of the recombinant enzyme. Appl. Environ. Microbiol..

[13] Collins T., Gerday C., Feller G. (2005). Xylanases, xylanase families and extremophilic xylanases. FEMS Microbiol. Rev..

[14] Garcia-Fraga B., Da S.A., Lopez-Seijas J., Sieiro C. (2014). Functional expression and characterization of a chitinase from the marine archaeon *Halobacterium salinarum* CECT 395 in *Escherichia coli*. Appl. Microbiol. Biotechnol..

[15] Eichler J. (2001). Biotechnological uses of archaeal extremozymes. Biotechnol. Adv..

[16] Uemori T., Sato Y., Kato I., Doi H., Ishino Y. (1997). A novel DNA polymerase in the hyperthermophilic archaeon, *Pyrococcus furiosus*: Gene cloning, expression, and characterization. Genes Cells.

[17] Shang Y.S., Zhang X.E., Wang X.D., Guo Y.C., Zhang Z.P., Zhou Y.F. (2010). Biochemical characterization and mutational improvement of a thermophilic esterase from *Sulfolobus solfataricus* P2. Biotechnol. Lett..

[18] Almeida R.V., Alqueres S.M.C., Larentis A.L., Rossle S.C. (2006). Cloning, expression, partial characterization and structural modeling of a novel esterasse from *Pyrococcus furious*. Enzym. Microbial. Technol..

[19] Cao H., Han H., Li G., Yang J., Zhang L., Yang Y., Fang X., Li Q. (2012). Biocatalytic Synthesis of Poly(delta-Valerolactone) Using a Thermophilic Esterase from *Archaeoglobus fulgidus* as Catalyst. Int. J. Mol. Sci..

[20] Liu J., Yu H., Shen Z. (2008). Insights into thermal stability of thermophilic nitrile hydratases by molecular dynamics simulation. J. Mol. Graph. Model..

[21] Kundu S., Roy D. (2010). Structure study of carboxylesterase from hyperthermophilic bacteria *Geobacillus stearothermophilus* by molecular dynamics simulation. J. Mol. Graph. Model..

[22] Abedi K.R., Abdul R.M., Basri M., Salleh A.B., Jacobs D., Abdul W.H. (2009). Molecular dynamics study of the structure, flexibility and dynamics of thermostable lipase at high temperatures. Protein J..

[23] Yin X., Li J.F., Wang J.Q., Tang C.D., Wu M.C. (2013). Enhanced thermostability of a mesophilic xylanase by N-terminal replacement designed by molecular dynamics simulation. J. Sci. Food. Agric..

[24] Mardanov A.V., Gumerov V.M., Slobodkina G.B., Beletsky A.V., Bonch-Osmolovskaya E.A., Ravin N.V., Skryabin K.G. (2012). Complete genome sequence of strain 1860, a crenarchaeon of the genus *Pyrobaculum* able to grow with various electron acceptors. J. Bacteriol..

[25] Yin D.L., Bernhardt P., Morley K.L., Jiang Y., Cheeseman J.D., Purpero V., Schrag J.D., Kazlauskas R.J. (2010). Switching catalysis from hydrolysis to perhydrolysis in *Pseudomonas fluorescens* esterase. Biochemistry.

[26] Ma J., Wu L., Guo F., Gu J., Tang X., Jiang L., Liu J., Zhou J., Yu H. (2013). Enhanced enantioselectivity of a carboxyl esterase from *Rhodobacter sphaeroides* by directed evolution. Appl. Microbiol. Biotechnol..

[27] Brumlik M.J., Buckley J.T. (1996). Identification of the catalytic triad of the lipase/acyltransferase from *Aeromonas hydrophila*. J. Bacteriol..

[28] Ginalski K. (2006). Comparative modeling for protein structure prediction. Curr. Opin. Struct. Biol..

[29] Eswar N., Webb B., Marti-Renom M.A., Madhusudhan M.S., Eramian D., Shen M.Y., Pieper U., Sali A. (2006). Comparative protein structure modeling using Modeller. Curr. Protoc. Bioinform..

[30] Wang Z., Li S., Sun L., Fan J., Liu Z. (2013). Comparative analyses of lipoprotein lipase, hepatic lipase, and endothelial lipase, and their binding properties with known inhibitors. PLoS One.

[31] Dundas J., Ouyang Z., Tseng J., Binkowski A., Turpaz Y., Liang J. (2006). CASTp: Computed atlas of surface topography of proteins with structural and topographical mapping of functionally annotated residues. Nucl. Acids Res..

[32] Park Y., Choi S.Y., Lee H. (2006). A carboxylesterase from the thermoacidophilic archaeon *Sulfolobus solfataricus* P1; purification, characterization, and expression. Biochim. Biophys. Acta.

[33] Hotta Y., Ezaki S., Atomi H., Imanaka T. (2002). Extremely stable and versatile carboxylesterase from a hyperthermophilic archaeon. Appl. Environ. Microbiol..

[34] Ogino H., Miyamoto K., Ishikawa H. (1994). Organic-solvent-tolerant bacterium which secretes organic-solvent-stable lipolytic enzyme. Appl. Environ. Microbiol..

[35] Jin P., Pei X., Du P., Yin X., Xiong X., Wu H., Zhou X., Wang Q. (2012). Overexpression and characterization of a new organic solvent-tolerant esterase derived from soil metagenomic DNA. Bioresour. Technol..

[36] Rao L., Xue Y., Zheng Y., Lu J.R., Ma Y. (2013). A novel alkaliphilic *bacillus* esterase belongs to the 13(th) bacterial lipolytic enzyme family. PLoS One.

[37] Kumar R., Singh R., Kaur J. (2013). Characterization and molecular modelling of an engineered organic solvent tolerant, thermostable lipase with enhanced enzyme activity. J. Mol. Catal. B Enzym..

[38] Leow T.C., Rahman R.N., Basri M., Salleh A.B. (2007). A thermoalkaliphilic lipase of *Geobacillus* sp. T1. Extremophiles.

[39] Tian J., Wang P., Gao S., Chu X., Wu N., Fan Y. (2010). Enhanced thermostability of methyl parathion hydrolase from Ochrobactrum sp. M231 by rational engineering of a glycine to proline mutation. FEBS J..

[40] Sharma P.K., Singh K., Singh R., Capalash N., Ali A., Mohammad O., Kaur J. (2012). Characterization of a thermostable lipase showing loss of secondary structure at ambient temperature. Mol. Biol. Rep..

[41] Radestock S., Gohlke H. (2008). Exploiting the link between protien rigidity and thermostability for data-driven protein engineering. Eng. Life Sci..

[42] Aurilia V., Rioux-Dube J., Marabotti A., Pezolet M., Auria S.D. (2009). Structure and dynamics of cold-adapted enzymes as investigated by FT-IR spectroscopy and MD. The case of an esterase from *Pseudoalteromonas haloplanktis*. J. Phys. Chem. B.

[43] Yu H., Huang H. (2014). Engineering proteins for thermostability through rigidifying flexible sites. Biotechnol. Adv..

[44] Bae E., Phillips G.J. (2005). Identifying and engineering ion pairs in adenylate kinases. Insights from molecular dynamics simulations of thermophilic and mesophilic homologues. J. Biol. Chem..

[45] Meng Y., Yuan Y., Zhu Y., Guo Y., Li M., Wang Z., Pu X., Jiang L. (2013). Effects of organic solvents and substrate binding on trypsin in acetonitrile and hexane media. J. Mol. Model..

[46] Bradford M.M. (1976). A rapid and sensitive method for the quantitation of microgram quantities of protein utilizing the principle of protein-dye binding. Anal. Biochem..

[47] Laemmli U.K. (1970). Cleavage of structural proteins during the assembly of the head of bacteriophage T4. Nature.

[48] Chen H., Wu J., Yang L., Xu G. (2014). Characterization and structure basis of *Pseudomonas alcaligenes* lipase’s enatiopreference towards D,L-menthyl propionate. J. Mol. Catal. B Enzym..

[49] Zheng X., Chu X., Zhang W., Wu N., Fan Y. (2011). A novel cold-adapted lipase from *Acinetobacter* sp. XMZ-26: Gene cloning and characterisation. Appl. Microbiol. Biotechnol..

[50] Gouet P., Courcelle E., Stuart D.I., Metoz F. (1999). ESPript: Analysis of multiple sequence alignments in PostScript. Bioinformatics.

[51] Laskowski R.A., MacArthur M.W., Moss D.S., Thornton J.M. (1993). Procheck: A program to check the stereochemical quality of protein structures. J. Appl. Cryst..

[52] Wiederstein M., Sippl M.J. (2007). ProSA-web: Interactive web service for the recognition of errors in three-dimensional structures of proteins. Nucleic Acids Res..

[53] Morris G.M., Huey R., Olson A.J. (2008). Using AutoDock for ligand-receptor docking. Curr. Protoc. Bioinform..

[54] Haq I.U., Khan M.A., Muneer B., Hussain Z., Afzal S., Majeed S., Rashid N., Javed M.M., Ahmad I. (2012). Cloning, characterization and molecular docking of a highly thermostable beta-1,4-glucosidase from Thermotoga petrophila. Biotechnol. Lett..

[55] Maraite A., Hoyos P., Carballeira J.D., Cabrera A.C., Ansorge-Schumacher M.B., Alcantara A.R. (2013). Lipase from *Pseudomonas stutzeri*: Purification, homology modelling and rational explanation of the substrate binding mode. J. Mol. Catal. B Enzym..

[56] Kerwin S.M. (2010). ChemBioOffice Ultra 2010 suite. J. Am. Chem. Soc..

[57] Yang X.Q., Liu J.Y., Li X.C., Chen M.H., Zhang Y.L. (2014). Key amino acid associated with acephate detoxification by *Cydia pomonella* carboxylesterase based on molecular dynamics with alanine scanning and site-directed mutagenesis. J. Chem. Inf. Model..

[58] Wang J., Wolf R.M., Caldwell J.W., Kollman P.A., Case D.A. (2004). Development and testing of a general amber force field. J. Comput. Chem..

[59] Jakalian A., Jack D.B., Bayly C.I. (2002). Fast, efficient generation of high-quality atomic charges. AM1-BCC model: II. Parameterization and validation. J. Comput. Chem..

