# Supplementary material for: Biochemical Characterization of a Carboxylesterase from the Archaeon Pyrobaculum sp. 1860 and a Rational Explanation of Its Substrate Specificity and Thermostability
Source: Int J Mol Sci. 2014 Sep 23;15(9):16885–910. doi: 10.3390/ijms150916885 (PMC4200780; doi:10.3390/ijms150916885)

Supplementary Information

Figure S1. Structure evaluation of the homology model. (**A**) Ramachandran plot of the P186_1588 model. The different color codes indicate most favored (red), generously allowed (dark yellow), additional allowed (light yellow), and disallowed (white) regions. 89.4% of the residues were in the most favored regions, 8.8% in additional allowed regions, and 0.0% in disallowed regions; (**B**) Structure evaluation by ProSA.

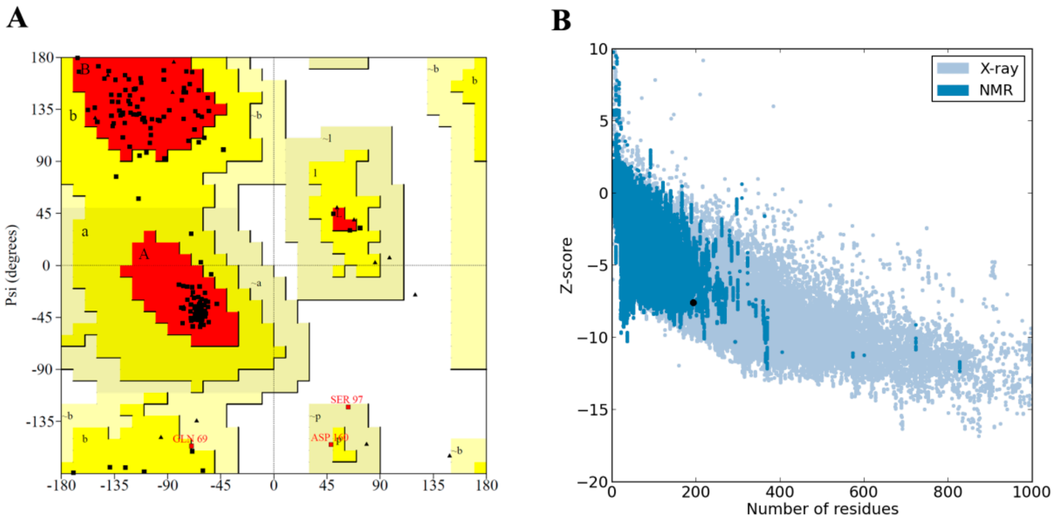

Figure S2. The two flexible regions (A1 and A2) in P186_1588. The A1 region consisted of residues 64–68, and was marked with light blue; The A2 region consisted of residues 144–156, and was marked with cyan. The catalytic residues (Ser97, Asp147 and His172) were marked with red.

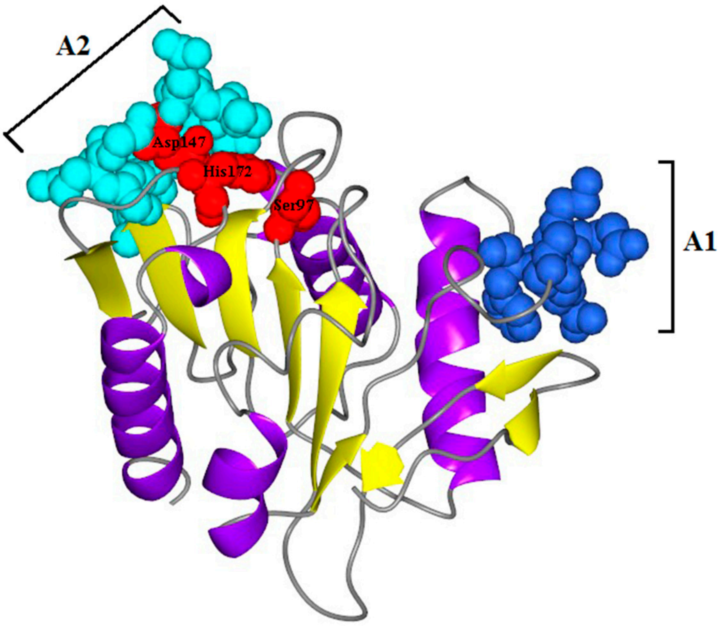

Supplement: Supplementary File 1 [file ijms-15-16885-s001.pdf]
